# Astrocytes Directly Influence Tumor Cell Invasion and Metastasis *In Vivo*

**DOI:** 10.1371/journal.pone.0080933

**Published:** 2013-12-04

**Authors:** Ling Wang, Stephanie M. Cossette, Kevin R. Rarick, Jill Gershan, Michael B. Dwinell, David R. Harder, Ramani Ramchandran

**Affiliations:** 1 Department of Pediatrics, Children’s Research Institute, Medical College of Wisconsin, Milwaukee, Wisconsin, United States of America; 2 Department of Physiology, Medical College of Wisconsin, Milwaukee, Wisconsin, United States of America; 3 Translational and Biomedical Research Center, Division of Hematology-Oncology, Medical College of Wisconsin, Milwaukee, Wisconsin, United States of America; 4 Department of Microbiology, Medical College of Wisconsin, Milwaukee, Wisconsin, United States of America; 5 Department of Obstetrics and Gynecology, Medical College of Wisconsin, Milwaukee, Wisconsin, United States of America; Northwestern University, United States of America

## Abstract

Brain metastasis is a defining component of tumor pathophysiology, and the underlying mechanisms responsible for this phenomenon are not well understood. Current dogma is that tumor cells stimulate and activate astrocytes, and this mutual relationship is critical for tumor cell sustenance in the brain. Here, we provide evidence that primary rat neonatal and adult astrocytes secrete factors that proactively induced human lung and breast tumor cell invasion and metastasis capabilities. Among which, tumor invasion factors namely matrix metalloprotease-2 (MMP-2) and MMP-9 were partly responsible for the astrocyte media-induced tumor cell invasion. Inhibiting MMPs reduced the ability of tumor cell to migrate and invade *in vitro*. Further, injection of astrocyte media-conditioned breast cancer cells in mice showed increased invasive activity to the brain and other distant sites. More importantly, blocking the preconditioned tumor cells with broad spectrum MMP inhibitor decreased the invasion and metastasis of the tumor cells, in particular to the brain *in vivo*. Collectively, our data implicate astrocyte-derived MMP-2 and MMP-9 as critical players that facilitate tumor cell migration and invasion leading to brain metastasis.

## Introduction

According to the “seed and soil” hypothesis [1], tumor cells spread to certain organs because of their specific microenvironment. Microenvironment of an organ comprises of cells, matrix and molecules secreted by cells in the environment. The metastasis of tumor cells to the brain is perhaps the critical final step in tumor progression. The astrocyte cells in the brain have been hypothesized previously to contribute to the altered brain microenvironment, and facilitate brain metastases [2], [3]. Anatomically, astrocytes are the one of the first host cell type that extravasating cancer cells encounter in the brain, and astrocytic foot processes influence microvascular integrity, which largely regulates transport of molecules and cells into the brain. Studies on brain metastasis from breast and lung cancer [4]–[8] showed that cancer cell arrests, extravasates, and invades brain parenchyma, activities associated with strong local activation of astrocytes. The activated astrocytes accumulate around the metastatic foci of tumor cells throughout the extravasation process [5], and are frequently observed in the vicinity of metastatic brain tumors in animal models and in human patients [4], [7]–[9]. To date, the role of astrocytes in the promotion of tumor growth and progression is poorly understood. One of the pressing questions in cancer biology is how do tumor cells migrate and invade the brain? We hypothesize here that tumor cell metastasis to the brain is influenced by astrocyte secretome [10], and astrocytes play a direct role in tumor metastasis. In this study, we tested the hypothesis that astrocytes directly influence tumor cell invasion and metastasis. Here, we report that rat neonatal and adult astrocytes secrete matrix metalloprotease enzymes (MMPs) that facilitate the invasion and metastasis of tumor cells *in vitro* (breast, lung and sarcoma) and *in vivo* (breast). Modulating the MMP activity prevents tumor cell invasion *in vitro* and metastasis *in vivo*. These findings implicate direct effects of astrocyte secretome on tumor cell growth and metastasis, and the involvement of the matrix molecules in this process.

## Materials and Methods

### Reagents

MMPs inhibitors ONO-4817, Marimastat and Batimastat were purchased from Tocris Biosciences. Neutralizing antibodies to MMP-2, MMP-9 and MMP-3 were purchased from Millipore, and the non-immune mouse IgG control was purchased from R&D. Antibodies to MMP-2 and MMP-9 that were used for both immunoprecipitation (IP) and western immunoblot (IB) were purchased from Santa Cruz. Recombinant human MMP-2 and MMP-9 were purchased from R&D. The MDA-MB-231.Br-Luc cells (brain seeking) were a kind gift from Dr. Balaraman Kalyanaraman [11], which were originally from Dr. Yoneda [12].

### Neonatal and Adult Hippocampal Astrocyte Culture

Primary cultures of neonatal hippocampal astrocytes were obtained as previously described [13]. Briefly, hippocampi were dissected from 1- to 2-day old Sprague Dawley rat pups and digested in papain (20 U/mL) and L-cystine (1.5×10^−4^ g/mL). The tissues were triturated, and the isolated cells were plated on 10 cm culture treated dishes at a density of approximately 1×10^6^ cells per dish in astrocyte growth media (DMEM containing 10% FBS, 1% Penicillin-Streptomycin). Primary adult hippocampal astrocytes were obtained from 10- to 12-week-old Sprague Dawley rats as previously described for adult rat neural cell cultures [14], [15], with some modifications. Briefly, the dissected and minced hippocampi were dissociated with gentle shaking for 20 min in papain (10 U/mL) that was not activated with L-cystine. The tissue was then centrifuged at 400 g for 5 min to remove the papain solution and re-suspended in astrocyte growth media supplemented with 0.1% Gentamicin and 2% B-27. The tissues were triturated in the same media, and the resulting cell suspension was filtered using a 40 µm cell strainer. The cell suspension was centrifuged at 400 g for 3 min and re-suspended in astrocyte growth media supplemented with 1% N-2. Isolated cells were plated at a density of approximately 3000 cells per mm^2^. Cell cultures were incubated at 37°C in an atmosphere of 5% CO_2_ in air. Culture media was changed three times per week. Confluent monolayers of neonatal astrocytes formed within 7–10 days after the initial plating, while adult astrocytes typically took 14–21 days. The primary cultures were maintained by passaging the cells when they were greater than 80% confluent.

### Tumor Cell Culture

The MDA-MB-231-Lu cells were cultured in DMEM (GIBCO), respectively. H2030 cells were cultured in RPMI 1640 medium (GIBCO). S180 cells were cultured in DMEM with 5 µg/mL blasticidin. All medium were supplemented with 10% FBS. To evaluate the effect of MMP inhibitors on the inhibition of MMP activity, astrocytes conditioned media (CM) were incubated with various concentrations of MMP inhibitors for 1 h at 37°C, and the resultant solutions were used for the assays. To investigate whether neutralizing anti-MMP-2 and anti-MMP-9 can block the functions of MMP-2 and MMP-9 in astrocyte CM, astrocytes were cultured in the presence of respectively, anti-MMP-2 or anti-MMP-9 antibodies, or both.

### Wound Healing Assay

5×10^5^ tumor cells/well were plated in a 24-well culture plate and incubated 24 h to form a monolayer. Monolayers of cells were scratched with a 200 µL Avant Premium Binding Pipet Tip (Midwest Scientific) and incubated in different CM in an incubation chamber-equipped Zeiss microscope system supplemented with heating (37°C) and 5% CO_2_ and imaged every 15 min for an indicated time period. Data are expressed as the mean ± S.D. of triplicate values.

### Boyden Chamber Migration Assay and Invasion Assay

Serum-starved tumor cells were seeded as 4×10^4^ cells/well in 500 µL of indicated medium into transwell inserts with 8 µm pores (Corning Incorporated). The transwell inserts were then inserted into a 24-well plate containing 750 µL of indicated CM and treatments. Cells were allowed to migrate at 37°C, 5% CO_2_ for an indicated time period. Cells were then fixed at 4% PFA at RT for >15 min, and were further stained for 5 min with crystal violet (Sigma) in 2% ethanol and then rinsed in water. The cells on the upper side of the inserts were removed with a cotton swab, and the cells on the lower side of the inserts that were counted under light microscopy. For invasion assay, transwell inserts were coated with 4 mg/mL of matrigel (BD Bioscience). Data are expressed as the mean ± S.D. of triplicate values.

### Heparin Agarose Binding Protein Purification

Heparin agarose was equilibrated with equilibration buffer (0.6 M NaCl/0.01 M Tris-HCl, pH 7.5). Astrocyte CM was then incubated with the heparin agarose at 4°C for 3 h, and the supernatant collected for invasion assays. Bound proteins were eluted with elution buffer (1.5 M NaCl/0.01 M Tris-HCl, pH 7.5), and collected for invasion assays.

### Ultrafiltration Protein Purification

30,0000 NMWL, 50,000 NMWL, and 100,000 NMWL Amicon ultra centrifugal filter devices (Millipore) were used to separate and concentrate tumor pro-migratory factors secreted by astrocytes. Astrocyte CM was added to the Amicon ultrafilter unit, and centrifuged at 2500×g, 4°C according to the manufacturers’ specifications. The filtrates were collected for related experiments.

### Gelatin Zymography

MMP-2 and MMP-9 enzymatic activity in the CMs were determined by SDS-PAGE gelatin zymography (See File S1). Three individual experiments were conducted with independent protein samples.

### Establishment of Astrocyte Secretome-induced Brain Metastatic MDA-MB-231-Luc Clone

To establish an astrocyte secretome-induced brain-seeking clone, the wildtype MDA-MB-231-Luc (MDA-MB-231WT-Luc) cells were grown in culture with astrocyte CM, and were recollected and grown in culture with astrocyte CM again. After five repeated passages in culture with the medium, the cells were used for *in vivo* experiments. To acquire an astrocyte secretome-induced MDA-MB-2.31 clone with depletion of MMP-2 and MMP-9, 50 µM ONO-4817 were added into astrocyte CM during the cell passaging. MDA-MB-231 astrocyte conditioned cells were routinely passaged every two days.

### Mouse Experiment

Male CB17/SCID mice (CB17/Icr-*Prkdc^scid^*/IcrCrl, Charles Rivers, Wilmington, MA, USA) (6–8 weeks) were selected for this study. After anesthesia using isoflurane, mice were inoculated with 2×10^5^ exponential growth phase tumor cells in a 100 µL volume via left ventricular injection. Ultrasound analysis was done using a VisualSonics Vevo 770 to visualize the injection needle in order to ensure left heart ventricle injection of the cells. Post injection, the tumor cell uptake was measured by bioluminescence using our previously published protocol [16], [17]. Briefly, tumors were imaged 4 min post 200 µL [15 mg/mL] intraperitoneal injection of D-luciferin (Xenogen Corporation). Isoflurane anesthetized mice were imaged at indicated time point using the Lumina IVIS-100 *in vivo* Imaging System (Xenogen Corporation). Regions of interest were created and measured as area flux, defined by radiance (photons per seconds per square centimeter per steradian) according to the manufacturer’s calibration (Xenogen Corporation). The MCW Animal Care and Use Committee approved all animal procedures under protocol AUA1022.

### Statistical Analyses

Differences in migration, invasion, and tumor burden were determined using an unpaired two-sided t test (p<0.05 was considered significant). Results are reported as mean ± SD. All *in vitro* experiments were performed at least three times.

## Results

### Conditioned Media from Astrocytes causes Tumor Cell Migration and Invasion

**Figure 1:**
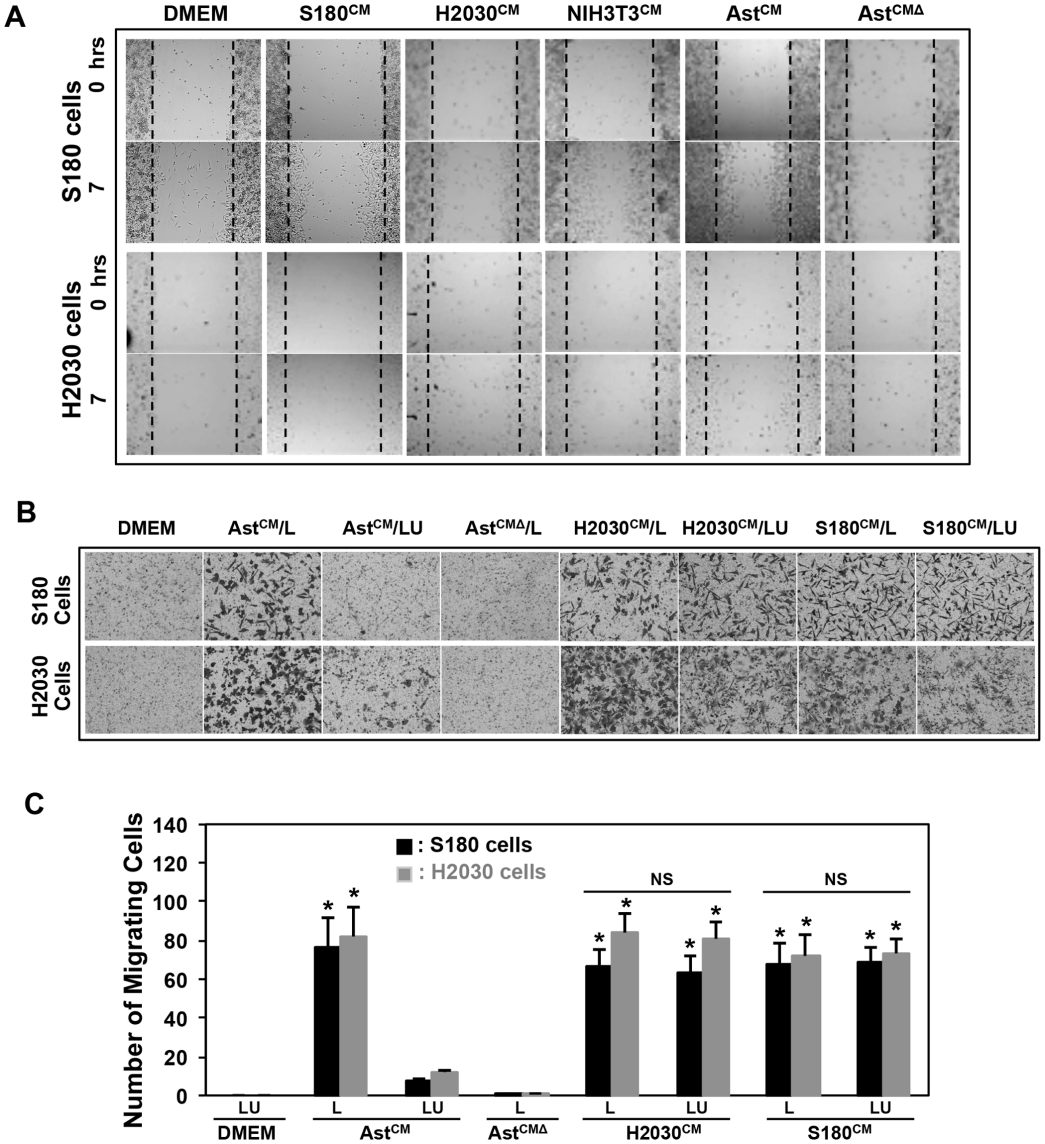
Effects of astrocyte secretome on tumor cell migration. (A) Representative images of S180 cell and H2030 cell migration. S 180 cells and H2030 cells were treated 8 h with different CMs from S180 cells, H2030 cells, NIH3T3 cells or astrocytes, respectively. (B) Representative images of S180 cell and H2030 cell transwell migration (Boyden chamber assay) for 5 h to examine the characteristics of astrocyte CM-induced tumor cell migration. (C) Quantitation of the number of migrated cells on the lower surface of the filter. L: lower chamber; LU: Lower chamber plus upper chamber. *p<0.01 compared with DMEM. For A and B, Ast^CM^: astrocyte CM; Ast^CMΔ^: astrocyte CM 120°C, 5–10 mins; H2030^CM^: H2030 cell CM; S180^CM^: S180 cell CM, NIH3T3^ CM^: NIH3T3 CM. Values are mean ± SD, n≥3.

Previous studies have shown the close apposition of tumor cells with astrocytes in the brain microenvironment [5]. To determine whether astrocytes influence tumor cells, we incubated sarcoma 180 (S180) cell line, lung adenocarcinoma H2030, and breast cancer MDA-MB-231 cell lines respectively in the conditioned culture media from two day old primary neonatal rat astrocyte (Ast) cells, and assessed migration and invasion potential of the tumor cells. The murine S180 cells are derived from a sarcoma cell line carried in Swiss Webster mice. S180 cells have been described to form highly aggressive cancers in lung, and grow in all strains of laboratory mice and rats due to β_2_-microglobulin deficiency, MHC class I destabilization, and lack of recognition by host cytotoxic T lymphocytes [18]. Lung cancer cells and breast cancer cells are prone to metastasis to the brain [19], thus rationalizing our choice. We compared the effects of primary neonatal rat astrocyte cells conditioned media (CM) or control DMEM to CMs from S180, H2030, and NIH3T3 cells on S180 cell and H2030 cell migration using wound closure assays that were carried out for 7 h in a 24-well format (Figure 1A). Time-lapse imaging was performed and still images were collected at 7 h. The CM from astrocytes (Ast^CM^) significantly closed the wound in S180, and H2030 cells at 7 h (Figure 1A), respectively. When astrocyte CM was heated to 120°C for 5–10 min (Ast-^CMΔ^), and added to S180 or H2030 cells, this effect of wound closure was abrogated (Figure 1A). This implies that heat sensitive components in astrocyte CM are responsible for wound closure of S180 and H2030 cells. Since motility associated with wound closure cannot distinguish between chemotaxis and chemokinesis, we performed Boyden chamber assays on S180 and H2030 cells. CM from astrocytes was added to the lower chamber (L), or in both the lower and upper chambers (LU), and the migration assay was performed for 5 h at 37°C. We noticed that S180 and H2030 cells migrated only when CM from astrocytes was present in the lower chamber when compared to both chambers (Figure 1B). In addition, heated CM from astrocytes (Ast^CMΔ^) did not induce migration of tumor cell types when placed in the lower chamber (Figure 1B). Cell quantification of H2030 cell migration clearly showed a significant difference (p<0.01) when CM from astrocytes was included in the lower chamber compared to both chambers (Figure 1C). To determine whether these effects were specific to astrocyte CM, we also performed tumor cell migration assays with CM from H2030 and S180 cells (Figure 1B). Both CM showed concentration-independent migration effects suggesting that this migration is likely chemokinetic in nature (Figure 1B). No statistical difference (p>0.05) in tumor cell motility was noted when either lower or both Boyden chambers contained H2030 or S180 CM (Figures 1B & 1C). Taking the wound healing and Boyden chamber migration data together, we conclude that astrocytes secrete a heat sensitive factor that induces chemotactic migration of tumor cells.

**Figure 2:**
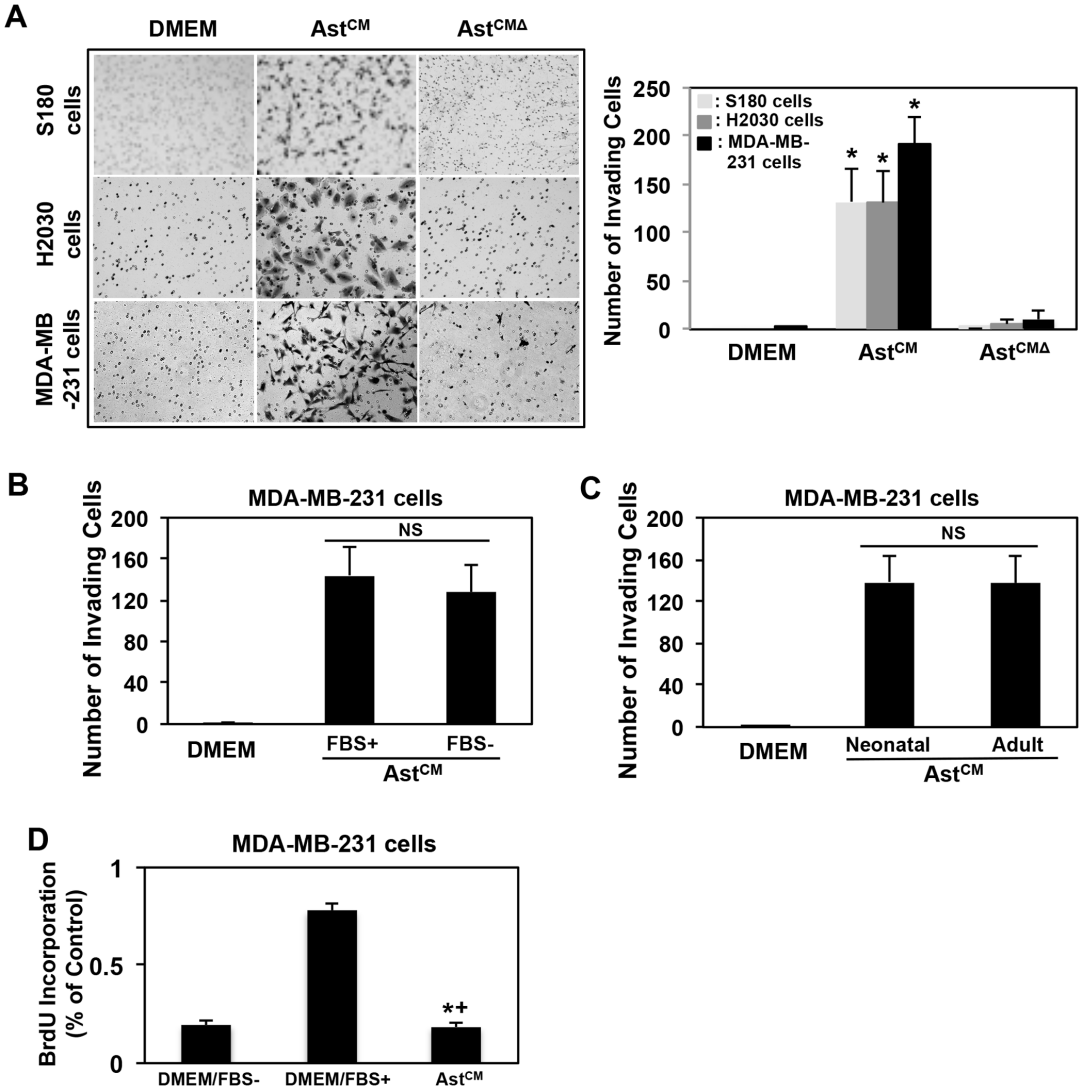
Effects of astrocyte secretome on cancer cell invasion. (A) Astrocyte CM induced tumor cell invasion. Matrigel invasion assays were performed with S180 cells (8 h), H2030 cells (8 h) and MDA-MB-231 cells (14 h) to examine the effects of astrocyte CM (Ast^CM^) and Astrocyte CM treated at 120°C, 5–10 mins (Ast^CMΔ^) on tumor cell invasion. *p<0.01 compared with DMEM or Ast^CMΔ^. Left panel is the representative images showing invaded cells on the lower surface of the filter, right panel is the quantity of the invaded cells; (B) Astrocyte CM-induced tumor cell invasion was independent of FBS. The CMs from astrocyte cultured in DMEM with or without 10%FBS (Ast^CM^/FBS+ or Ast^CM^/FBS−) were applied to invasion assay of MDA-MB-231 cells (14 h). NS: no significance. (C) Adult astrocyte CM induced tumor cell invasion. MDA-MB-231 cells were applied to neonatal or adult astrocyte CM (Ast^CM^/Neonatal or Ast^CM^/Adult), respectively, for invasion assay (14 h). NS: no significance. For A, B and C. values are mean ± SD, n≥3. (D) Effects of astrocyte CM on MDA-MB-231 cell proliferation. The BrdU assay was used to determine tumor cell proliferation. MDA-MB-231 cells cultured in astrocyte CM for 24 h, cells cultured in DMEM/FBS− or DMEM/FBS+ were used as control. The data are expressed as mean ± SD. The experiments were performed in triplicate. *p<0.01 compared with DMEM/FBS.

Since invasion is a critical component of tumor metastasis, we further tested the role of astrocytes CM in the invasion of sarcoma, lung and breast cells. CM from astrocytes in the lower chamber significantly induced matrigel invasion of S180, H2030 and MDA-MB-231-Luciferase (MDA-MB-231-Luc) cells compared with cells treated with Ast^CMΔ^ or DMEM (Figure 2A), which is consistent with the above data showing the effects of astrocyte CM on tumor cell migration. To determine whether FBS in astrocyte CM contributed to invasion we cultured astrocytes in DMEM without adding FBS (astrocyte^CM^/FBS−). The astrocyte^CM^/FBS− also induced MDA-MB-231 cell invasion (Figure 2B) compared to the CM from astrocytes cultured in DMEM with FBS (astrocyte^CM^/FBS+). This result suggests that astrocytes secrete factor(s) responsible for the tumor cell invasion process, and is independent of serum. To examine the influence of the adult astrocyte CM on tumor cell invasion, MDA-MB-231 cells were subjected to invasion assay with adult astrocyte CM (astrocyte^CM^/Adult) induction. Our results showed that adult astrocyte CM induced a similar invasion response compared to neonatal astrocyte CM (Figure 2C), which suggests the observed effect of astrocytes is independent of their age. Bromodeoxyuridine (BrdU) assay (See File S1) and Caspase-Glo® 3/7 Assay were also performed in MDA-MB-231 cells with astrocyte CM. Astrocyte CM-conditioned tumor cell did not proliferate (Figure 2D), and the apoptosis results were equivocal. Collectively, our data suggest that astrocytes secrete heat sensitive factor(s) that contributes to breast, lung and sarcoma tumor cell migration and invasion in vitro.

### Heat Sensitive Factor Secreted by Astrocytes is Neither EETs Nor Binds to Heparin

**Figure 3:**
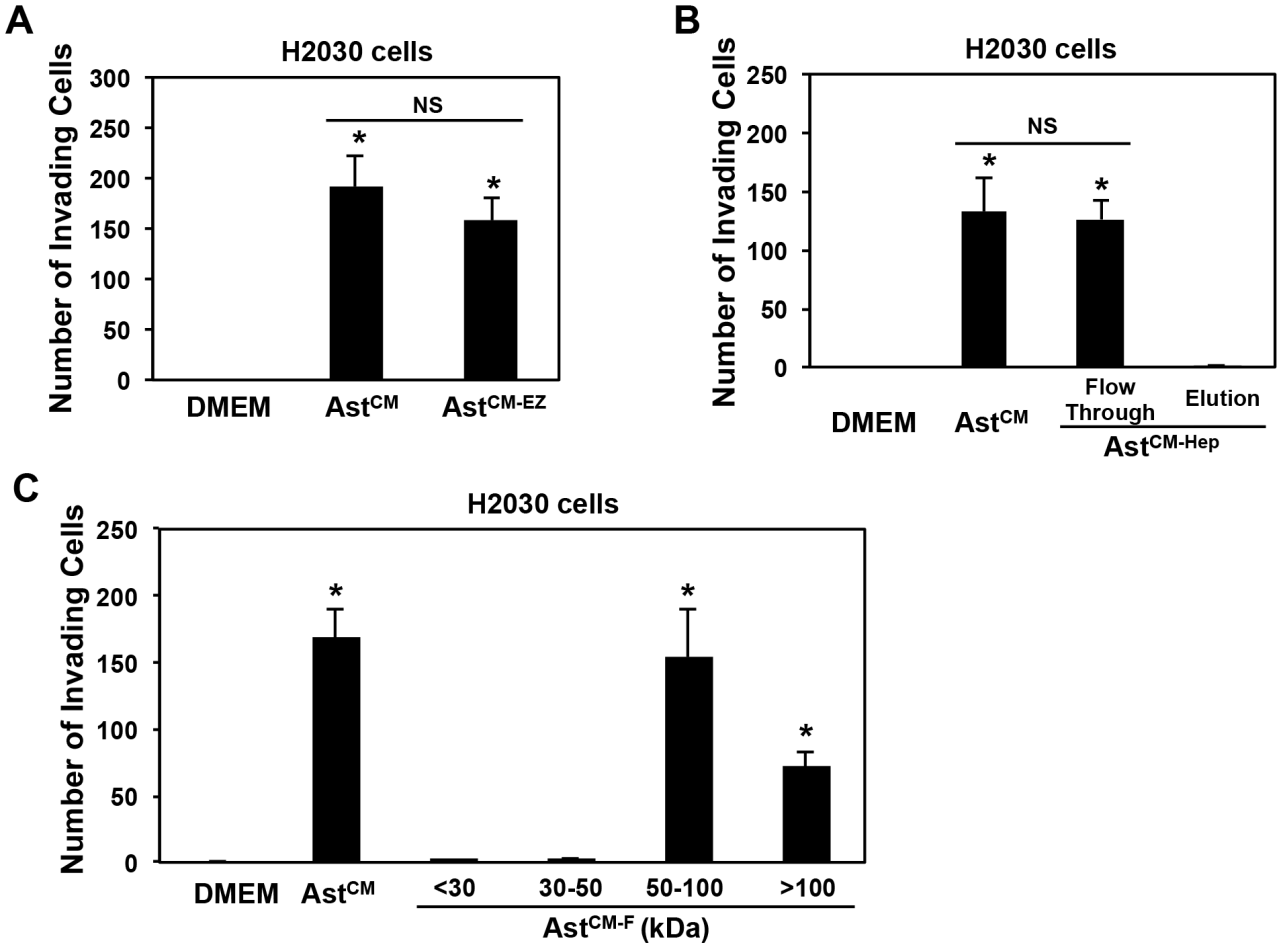
Characteristics of astrocyte-secreted components on lung cancer cell invasion. (A) Effects of EET-inhibitor EET-EZ on astrocyte CM-induced H2030 cell invasion. H2030 cells were used for invasion assay for 8 h in 10 µM EETs antagonist EET-EZ treated astrocyte CM (Ast^CM-EZ^). *p<0.01 compared with DMEM. NS: no significance; (B) Effects of heparin binding proteins on H2030 cell invasion: Astrocytes CM incubated with heparin agarose beads for 3 h (Ast^CM-Hep^), the resulted flow through or elution were applied to invasion assay, respectively, for 8 h. *p<0.01 compared with DMEM. NS: no significance; (C) H2030 cell invasion in astrocyte CM fractions. Astrocyte CMs from ultrafiltration cut-off (Ast^CM-F^) were supplied to H2030 cell invasion assay (8 h). *p<0.01 compared with DMEM. For A, B and C, values are mean ± SD, n≥3.

Previous work has implicated the role of epoxyeicosatrienoic acids (EETs) (lipids that are heat sensitive and secreted by astrocytes) in promoting processes such as endothelial cell mitogenesis and morphogenesis, processes that are involved in tumor metastasis [20]. To eliminate EETs as a probable astrocyte heat sensitive factor that promotes tumor cell migration, we treated astrocyte CM with the EETs inhibitor EET-EZ. We then compared the S180 and H2030 cells migration response to EET-inhibitor treated or non-treated astrocyte CM in a Boyden chamber migration assay (Figure S1A). No qualitative difference in tumor cell migration response was noted between the EETs-treated astrocyte CM and untreated astrocyte CM (Figure S1A). Furthermore, we tested the invasion response of H2030 cells to EET-inhibitor treated and non-treated astrocyte CM, and found similar response in cell invasion assay (Figure 3A). These results imply that EETs are less likely to play a significant contribution to the tumor pro-migratory or pro-invasive property observed in astrocytes CM.

To determine the identity of this factor(s), we incubated astrocyte CM with heparin agarose beads for 3 h rationalizing that growth factors that bind heparin will be unavailable for inducing pro-migratory or pro-invasive activity. Interestingly, the heparin-treated astrocyte CM continues to promote H2030 and S180 tumor cell migration (Figure S1B), and invasion (Figures 3B and S1C). Further, bound eluates from the heparin-column failed to stimulate either migration or invasion (Figures 3B & S1C). This data suggests that the heat sensitive factor(s) is less likely to bind to heparin, which eliminates the majority of growth factors that bind to heparin as candidate factors that promote tumor cell migration and invasion.

### Astrocyte-secreted Heat Sensitive Factor Size is Greater than 50 kDa

To investigate the size of the heat sensitive factor(s) secreted by astrocytes, we concentrated astrocyte CM by ultrafiltration through <30 kDa, 30–50 kDa, 50–100 kDa and 100 kDa membrane filter cut-offs. Ultrafiltration membranes are used both to purify material passing through the filter, and also to collect material retained by the filter. Materials significantly smaller than the pore size pass through the filter and materials larger than the pore size are retained by the filter. CMs that were concentrated using the ultrafiltration technique were placed in the lower Boyden chamber for an invasion assay of H2030 and S180 cells as described earlier. As shown in Figures 3C (H2030 cells) and S1D (S180 cells), astrocyte CM from <30 kDa, and 30–50 kDa cut-off showed no effect on H2030 and S180 cell invasion, whereas CM from 50–100 kDa ultra filter showed similar invasion of H2030 and S180 cells, and CM from >100 kDa showed some invasion of H2030 and S180 cells. We focused on the 50–100 kDa fraction for further studies since this fraction contained the majority of the tumor promoting activity. These results suggest that the size of the heat sensitive factor secreted by astrocytes is greater than 50 kDa.

### MMP-2/-9 and -3 Partly Mediate the Astrocyte Secretome-induced Tumor Cell Invasion

**Figure 4:**
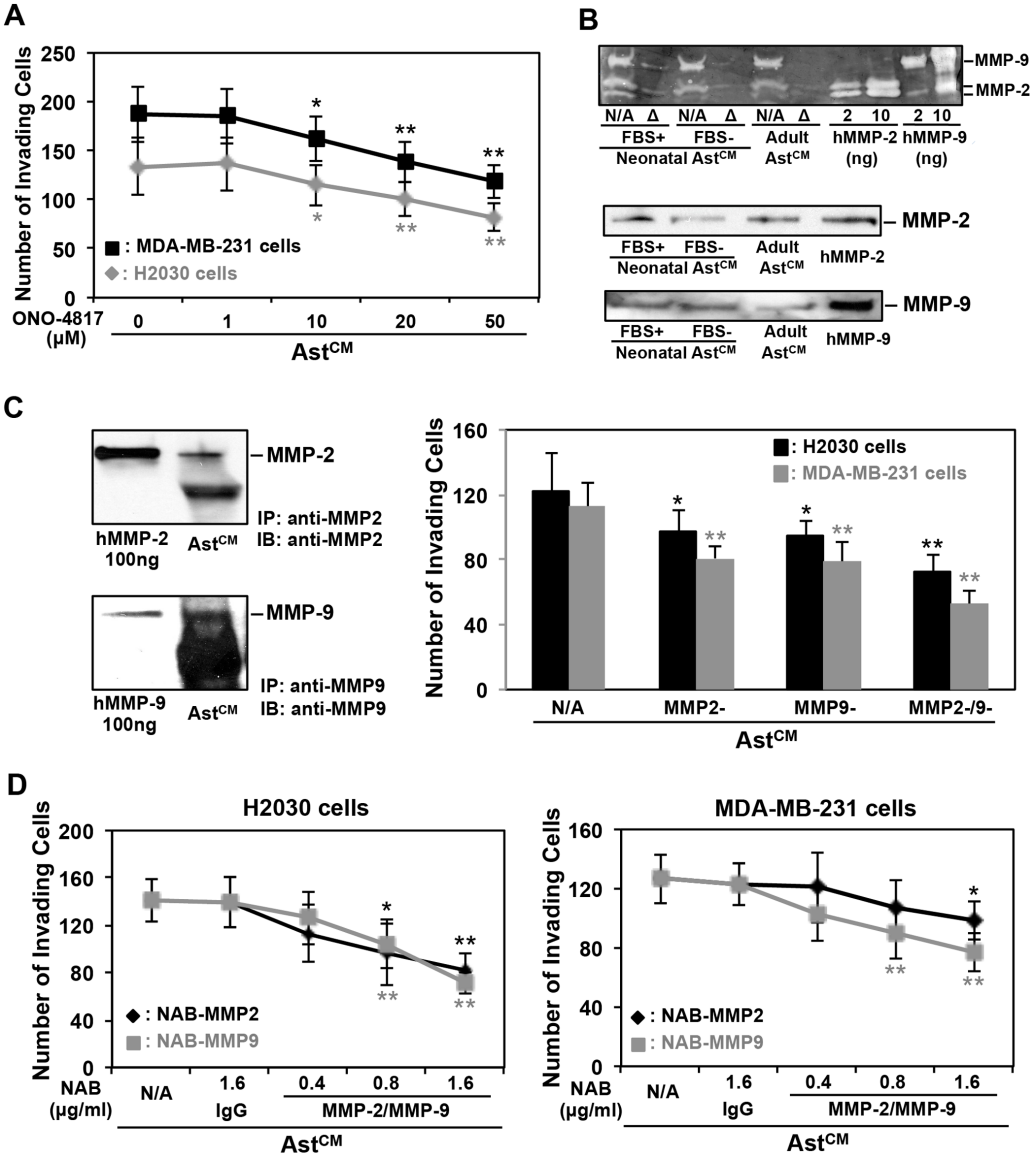
Effects of astrocyte-secreted MMP-2 and MMP-9 on tumor cell invasion. (A) Effects of MMP inhibitor ONO-4817 on astrocyte CM-induced cancer cell invasion. Invasion assays were performed with H2030 cells (8 h) and MDA-MB-231 cells (14 h) in astrocyte CM (Ast^CM^) pre-treated with different dosage of ONO-4718 for 45 min, respectively. (B) Determination of the astrocyte-secreted MMP-2 and MMP-9. Upper panel is the gelatin zymography to show MMP-2 and MMP-9 enzyme activity in neonatal and adult astrocyte CM; lower panel is the western blotting to show MMP-2 and MMP-9 proteins in astrocyte CM; (C) Pull-down of MMP-2 or MMP-9 or both to further examine astrocyte CM-induced tumor cells invasion. Astrocyte CMs were immunoprecipitated MMP-2/-9 with respective antibodies, the resulted medium was used for invasion assays; (D) Effects of blocking MMP-2 and MMP-9 function on cancer cell invasion. Astrocytes were cultured in DMEM with indicated concentration of MMP-2 and MMP-9 neutralizing antibodies, respectively, and the resulted media were submitted to invasion assay. For A, C and D, *p<0.05 and **p<0.01 when compared with Ast^CM^. Values are mean ± SD, n≥3.

By analyzing the molecular weight of the known astrocyte secretome (Table S1) from the literature, the majority of proteins were excluded because their molecular weight is less than 50 kDa. However, matrix metalloprotease (MMP) enzymes secreted by astrocytes are in the molecular weight (MW) range of 50–100 kDa, and are known to play an important role in tumor invasion and angiogenesis by mediating degradation of the extracellular matrix, which promotes metastasis [21]. We investigated whether MMPs are involved in the astrocyte secretome-induced tumor cell invasion. Our first approach was a chemical biology approach where astrocyte CMs treated separately with broad-spectrum MMP inhibitors ONO-4817, Batimastat and Marimastat were tested on tumor cells in a wound closure assay. MMP inhibitors specifically ONO-4817 showed inhibitory effects on the astrocyte CM-induced S180 cell migration (Figure S2A). Concentrations of ONO-4817 were determined based on the half inhibition concentration (IC50) that shows the lowest MMP-2 and MMP-9 IC50 for ONO-4817 (TOCRIS Bioscience). When ONO-4817 treated astrocyte CM was applied to invasion assay, we found that H2030 cell (Figure 4A), MDA-MB-231 (Figure 4A) and S180 (Figure S2B) cell invasion were inhibited in a dose-dependent manner. A recent study showed that NFATc3 promotes Ca^2+^-dependent MMP3 expression in astroglial cells [22], we therefore examined whether MMP-3 is also involved in the astrocyte CM-induced MDA-MB-231 cell invasion. MMP-3 in astrocyte CM was immunoprecipitated (IP’d) with MMP-3 antibody, and the resultant CM was tested for invasion activity. We found that MMP-3 was partially involved in astrocyte CM-induced breast cancer cell invasion but to a lesser extent compared with either MMP-2 or MMP-9 (Figure S2C).

To test whether MMPs by themselves serve as chemoattractant we added purified human MMP-2, MMP-3 and MMP-9 proteins to the lower chamber in the Boyden chamber invasion assay. Purified MMP-2, MMP-3 or MMP-9 proteins were unable to induce MDA-MB-231 cells invasion (Figure S2D). These results suggest that MMPs do not act as chemoattractants. Since MMP-2 and MMP-9 showed more tumor cell invasive activities than MMP-3, we focused on MMP-2 and -9 moving forward.

To determine the MMP profile in astrocyte CM, we performed gelatin zymography (See File S1) for MMP-2 and MMP-9, well known to correlate with the invasive and metastatic potentials of various cancers [23], [24]. As shown (Figure 4B, upper panel), we observed three clear bands at the location where purified human MMP-2 and MMP-9 proteins in neonatal (with or without adding FBS) and adult astrocyte CM migrated on the gel, which corresponds to the molecular weight of MMP-9 (92 kDa for latent and 82 kDa for active form) and MMP-2 (72 kDa for latent and 62 kDa for active form). Interestingly, all bands disappeared when the astrocyte CM was heated at 120°C for 5–10 min (Figure 4B, upper panel Δ lane). We also performed western blotting analysis, and found that anti-MMP2 and anti-MMP9 antibodies detected MMP-2 and MMP-9 proteins, respectively, in both neonatal (with or without adding FBS) and adult astrocyte CM (Figure 4B, lower panel). Together, these results suggest that astrocytes are able to produce and secrete active MMP-2 and MMP-9 enzymes.

To determine whether MMP-2 or MMP-9 in the astrocyte CM were involved in inducing cancer cell invasion, we IP’d MMP-2, MMP-9 or both in astrocyte CM with specific respective antibodies (Figure 4C, left panel), and performed an invasion assay with the astrocyte CM depleted of the MMPs. As shown (Figure 4C, right panel), H2030 and MDA-MB-231 cells exposed to astrocyte CM depleted of MMP-2 or MMP-9 showed a significant decrease in the ability for cell invasion. We also blocked MMP-2 and MMP-9 functions with MMP-2 and MMP-9 neutralizing antibodies, respectively, and found that astrocyte CM containing neutralizing antibodies reduced its ability to induce both breast and lung cancer cell invasion (Figure 4D). This reduction in cancer cell invasion was dependent on the dose of antibodies used (Figure 4D). We observed significant reduction in cell invasion (approximately 50%) using 1.6 µg of anti-MMP-2 and anti-MMP-9 antibodies together in H2030 cells, as well as in MDA-MB-231 cells compared to the IgG control in both cells (Figure 4D). Taken together, these *in vitro* results suggest that astrocyte secreted MMP-2 and MMP-9 proteins partially mediate tumor cell invasion.

### Astrocyte-conditioned Tumor Cells are Metastatic *in vivo*

**Figure 5:**
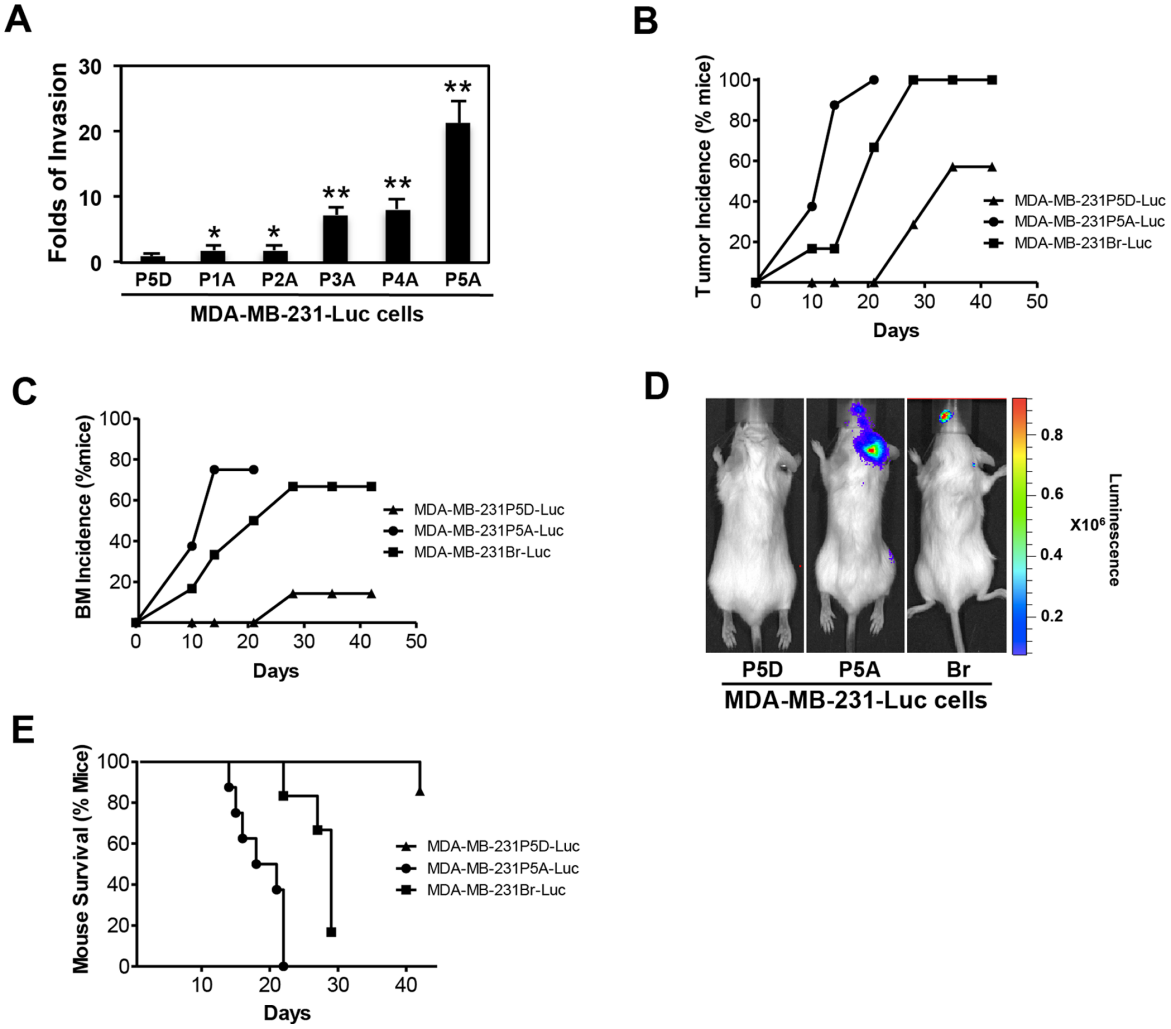
Astrocyte secretome-induced tumor cells metastasize to the brain *in vivo*. MDA-MB-231-Luc cells were sequentially passaged in astrocyte CM or DMEM. (A) Matrigel invasion of the each passage cells (MDA-MB-231P1-5A-Luc and MDA-MB-231P5D-Luc) was examined by invasion assay. *p<0.05 and **p<0.01 compared with MDA-MB-231P5D-Luc cells, values are mean ± SD, n≥3; (B–E) Passage 5 MDA-MB-231P5A-Luc (n = 8), MDA-MB-231P5D-Luc cells (n = 7) and brain homing MDA-MB-231Br-Luc cells (n = 6) were inoculated into mice via the intra-cardiac route, respectively. Tumor metastasis was measured by bioluminescent imaging. (B) Tumor incidence; (C) BM incidence; (D) Representative images show the brain metastasis; (E) Percentage of mice survival.

To establish an *in vivo* brain metastasis model, we sequentially passaged MDA-MB-231-Luc cells in astrocyte CM culture. Each serially passaged MDA-MB-231-Luc cells were subjected to an astrocyte CM-induced matrigel invasion assay. Intriguingly, each subsequent passage of MDA-MB-231-Luc cells in astrocyte CM increased the invasion competence several fold with passage 5 cells showing an approximate 20-fold increase in invasion compared to passage 1 cells (Figure 5A). We used the 5^th^ generation of astrocyte CM passaged MDA-MB-231-Luc (MDA-MB-231P5A-Luc) cells as comparison to the 5^th^ generation of DMEM passaged MDA-MB-231-Luc (MDA-MB-231P5D-Luc) cells for our *in vivo* experiments. We injected CB17/SCID mice with 2×10^5^ MDA-MB-231P5D-Luc cells, MDA-MB-231P5A-Luc cells and brain homing MDA-MB-231Br-Luc cells via the intra-cardiac route, respectively. The MDA-MB-231Br-Luc cells were previously generated by serially passaging cells in mice [12]. We monitored tumor-injected mice using biophotonic imaging at day 0, 2, 4, 6, 8, 10, 12, 14, 21, 28, 35 and 42 of inoculation. 40% of mice injected with MDA-MB-231P5A-Luc cells showed tumor incidence at day 10 compared with 20% of mice injected with MDA-MB-231.Br-Luc cells (Figure 5B). By day 14, 87.5% of mice injected with MDA-MB-231P5A-Luc cells showed tumor incidence, which was observed by day 25 in mice injected with MDA-MB-231.Br-Luc cells indicating a ten day delay in incidence rate for the brain homing tumor cells (Figure 5B and Table S2). The incidence of brain metastasis however, was clearly much higher by day 14 in mice injected with MDA-MB-231P5A-Luc cells (75%) compared to 33.33% for mice injected with MDA-MB-231Br-Luc cells (Figure 5C and Table S2). Also, at the same time point (day 14), mice injected with MDA-MB-231P5A-Luc cells showed more tumor cells in the brain than mice injected with MDA-MB-231Br-Luc cells (Figure 5D and Table S2). Consistent with those data, the survival of mice was greatly decreased and reached 0% by day 21 for mice injected with MDA-MB-231P5A-Luc cells, while compared to 100% survival was noted for mice injected with MDA-MB-231Br-Luc cells (Figure 5E). All images of mice are shown in Figure S3. This data collectively suggests that the astrocyte-conditioned tumor cells showed a higher incidence of tumor cells in the brain leading to early mortality.

### Astrocyte-secreted MMP-2/-9 Mediate Astrocyte CM-induced Breast Tumor Brain Metastasis

**Figure 6:**
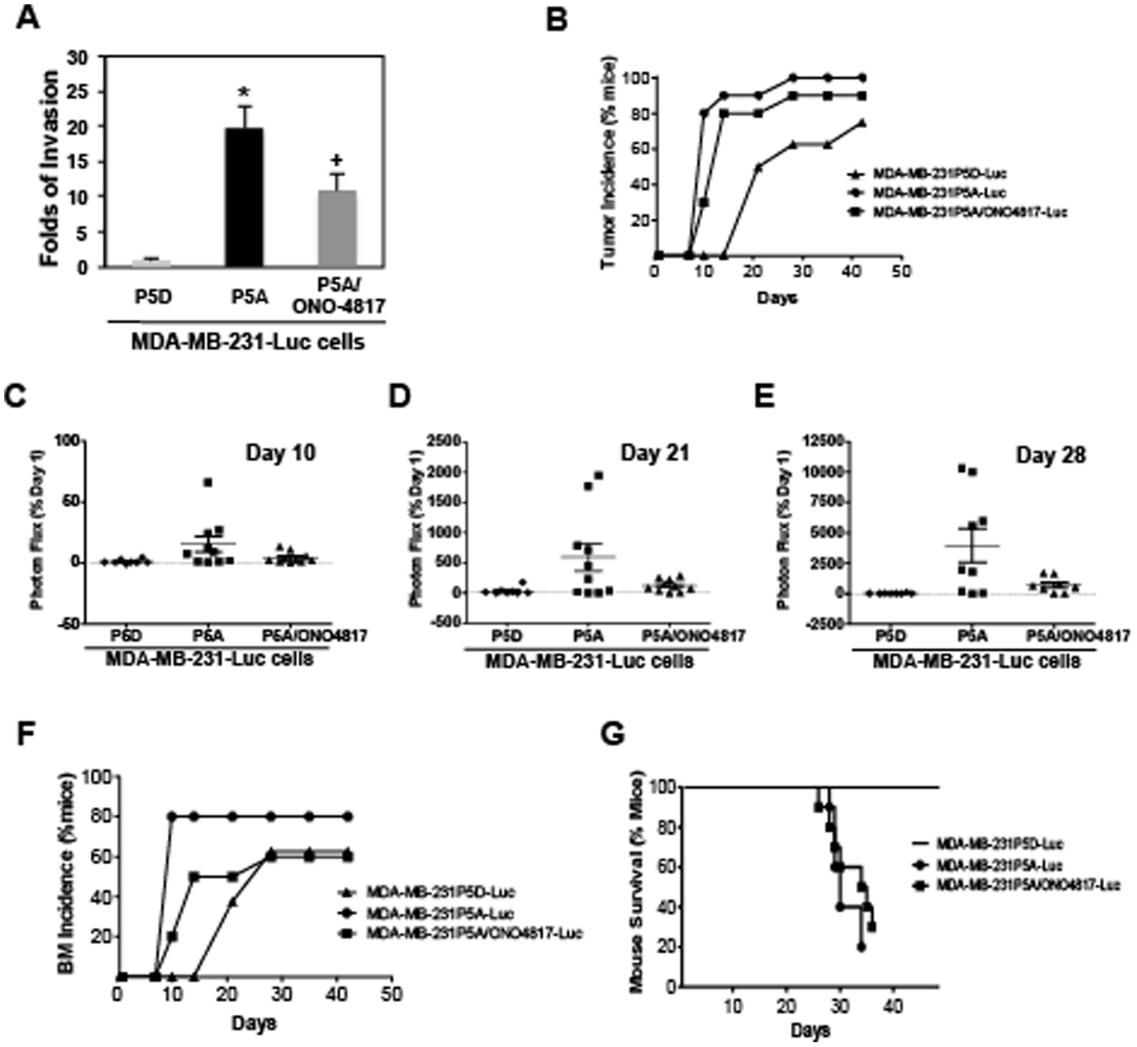
Inhibition of astrocyte-secreted MMP-2 and MMP-9 partially reduce astrocyte secretome-induced breast cancer brain metastasis formation. MDA-MB-231-Luc cells were sequentially passaged in astrocyte CM treated without or with the MMP inhibitor ONO-4817, and passage 5 cells (MDA-MB-231P5A/ONO4817-Luc) were used for (A) matrigel invasion assay to compare the metastasis ability of the passage 5 cells (MDA-MB-231P5A/ONO4817-Luc to MDA-MB-231P5A-Luc). *p<0.01 compared with MDA-MB-231P5D-Luc, ^+^p<0.01 compared with MDA-MB-231P5A-Luc. Values are mean ± SD, n≥3; (B–G) the passage 5 cells [MDA-MB-231P5A-Luc (n = 10), MDA-MB-231P5A/ONO4817-Luc (n = 10), and the MDA-MB-231P5D-Luc cells (n = 8)] were inoculated into mice via the intra-cardiac route, respectively. Tumor metastasis was measured by bioluminescent imaging. (B) Tumor incidence; (C–E) The normalized photon flux at day 10, 21 and 28; (F) BM incidence; (G) Percentage of mice survival.

On the basis that astrocyte-secreted MMP-2 and MMP-9 partially mediated tumor cell invasion *in vitro*, we investigated whether astrocyte-secreted MMP-2 and MMP-9 are involved in astrocyte CM-induced brain metastasis. We passaged MDA-MB-231-Luc cells 5 times in astrocyte CM treated with the MMP inhibitor ONO-4817 to obtain MDA-MB-231P5A/ONO4817-Luc cells. When these cells were tested in invasion assay, we observed a 50% reduction in tumor invasion activity when compared to the MDA-MB-231P5A-Luc cells (Figure 6A). MDA-MB-231P5A/ONO4817-Luc cells were further investigated for their brain metastasis ability. Because injection of 2×10^5^ cells induced rapid mortality of mice in MDA-MB-231P5A-Luc cells in our first set of *in vivo* experiments (Figure 5), we injected half as much (1×10^5^ cells) in the second *in vivo* experiment. MDA-MB-231-P5D-Luc, MDA-MB-231P5A-Luc and MDA-MB-231P5A/ONO4817-Luc cell lines were injected via the intra-cardiac route. Biophotonic imaging showed while both MDA-MB-231P5A-Luc and MDA-MB-231P5A/ONO4817-Luc cells developed more rapid and severe tumor metastasis when compared to the control MDA-MB-231-P5D-Luc cells (Figures 6B, 6C, 6D & S4B, Table S3), MDA-MB-231P5A/ONO4817-Luc cells showed relative slower development of tumor metastasis than MDA-MB-231P5A-Luc cells (30% vs. 80% at day 10) (Figure 6B and Table S3), and also less severe tumor metastasis compared to MDA-MB-231P5A-Luc cells (Normalized photon flux to day 1 are 4.15 vs. 15.1 at day 10, 118.8 vs. 595.1 at day 21, and 708.9 vs. 3970.4 at day 28) (Figures 6C, D, E & S4B). The decreased normalized photon flux from day 28 was caused by the death of mice (Figure S4B and Table S3). More importantly, mice inoculated with MDA-MB-231P5A/ONO4817-Luc cells developed slower and less severe brain metastasis (60% less at day 10, 30% less at day 14, and 20% less from day 28 till 42) than mice inoculated with MDA-MB-231P5A-Luc cells (Figure 6F and Table S3). Consistent with tumor incidence and tumor brain metastasis incidence, the survival of mice was partially increased in mice inoculated with MDA-MB-231P5A/ONO4817-Luc cells compared to the mice inoculated with MDA-MB-231P5A-Luc cells (80% vs. 50% at 28 days, 40% vs. 20% at day 35, and 30% vs. 20% at day 42 (Figure 6G and Table S3). All images of mice are shown in Figure S4A. Together, our data provide evidence that astrocyte secreted MMP-2 and MMP-9 partially mediate astrocyte secretome induced breast cancer metastasis to brain.

## Discussion

Data from our lab provides evidence that the astrocytic secretome influences the local microenvironment, thereby facilitating migration and invasion of breast and lung cancer cells synonymous with metastatic behavior. Because metastasis to the brain is a critical determinant of morbidity, it is vitally important to understand the mechanisms of brain metastasis, thereby facilitating the development of molecular targeted agents that can prevent brain metastasis. Here, we demonstrate that astrocyte-conditioned tumor cells display highly invasive and metastatic behavior *in vitro* and *in vivo.* MMP-2 and -9 are two factors in the astrocyte secretome that are partially responsible for this response, and blocking MMP-2 and -9 proteins partially prevents the invasion and metastasis of tumor cells *in vitro* and *in vivo*.

Clinical observations of cancer patients and studies with experimental rodent tumors have concluded that certain tumors produce metastases to specific organs independent of vascular anatomy, and rate of blood flow [25]–[27]. Indeed, more than 100 years ago, British physician Steven Paget had argued that metastasis was not a random process, but that specific cancers spread only to certain organs – those that served as “soil” for specific cancer “seeds” [28]. The brain provides a unique environment due to the BBB, a restrictive barrier that is comprised of endothelial cells (ECs) connected by tight junctions, the basement membrane and astrocytic end-feet processes. The BBB protects the brain against the entry of most drugs and invasion by microorganisms [29]. However, injection of human tumor cells into athymic mice revealed that some types of tumor cells, such as carcinomas of the breast, kidney, lung and melanoma were able to cross the BBB to seed in the brain parenchyma [30], [31] while other primary neoplasms such prostate or colorectal cancer rarely cross the BBB, suggesting that metastasis of circulating tumor cells to the brain is not a simple “diffusion” process. Thus tumor cell metastasis to the brain is influenced by specific affinity of tumor cells (the ‘seed’) for the brain (the ‘soil’), which are potentially catalyzed by trophic factors [10]. In this study, we have identified some of those trophic factors secreted by astrocytes in the soil.

In general, astrocytes secrete a wide variety of proteins [32], [33]. Among them IL-6, TGF-β and IGF-1 might contribute to the development of brain metastasis in breast cancer cells [34]. Our filter-cut off studies eliminate most of the cytokines, and heparin agarose binding studies eliminate the remainder of growth factors that have affinity for heparin. Since filter cut off studies implicate proteins in the 50–100 kDa range to possess the tumor invasive activity, we anticipate that some of this activity resides in MMPs, more specifically MMP-2 (72 and 62 kDa), -9 (92 and 82 kDa) and -3 (54 kDa) because the MW of these MMPs fall in the range of 50–100 kDa. Indeed, MMP-2 and MMP-9 were detected by gelatin zymography and western blotting in this study, which are consistent with those observed by Gottschall *et al* where they reported that 1-day-old rat pup brain astrocytes produced MMP-2 after 24 h culture under basal conditions [35]. Also, MMP-2 and -9 have been observed in secretory vesicles in astrocytes [36] implying that some stimulus is responsible for this secretion. Interestingly, astrocytes stimulated with lipopolysaccharide, interleukin-1 alpha or beta, or tumor necrosis factor-alpha for 24 h induce MMP-2 and MMP-9 expression [35], of which MMP-9 is known to be involved in an MDA-MB-435 brain metastasis model [5]. Strong up-regulation of MMP-9 protein associated with astrocytes was observed in the immediate vicinity of extravasating cancer cells [5], and is known to promote growth of primary brain tumors by releasing vascular endothelial growth factor (VEGF) sequestered in the surrounding matrix [37]. Our data here implicates both MMP-2 and -9 secreted by astrocytes in the tumor invasion and metastasis process. Blocking MMP-2 and MMP-9 with MMP inhibitors or antibodies against MMP-2 or/and MMP-9 partially rescues the invasion and metastasis effects thus providing direct evidence for the role of astrocyte-secreted MMP-2 and MMP-9 in tumor invasion and brain metastasis. These data collectively argue for a role for MMP-2 and MMP-9 secreted by astrocytes in tumor invasion and metastasis. Further, our data imply that MMP-2 and -9 are not the only activities in the astrocyte CM that promote tumor cell invasion and metastasis because MMP chemical inhibition and blocking/neutralizing studies only attenuated about 50% of the tumor cell invasion. Additional activities in the astrocyte CM (other than MMPs) that contribute to tumor cell invasion are a subject of active investigation in our laboratory. In addition, we also observed that CM from astrocytes induced much less invasion of colon HCT116 tumor cells and almost didn’t induce invasion of non-metastasis breast MCF-7 tumor cells (Figure S5), which suggests that preferential substrates for MMPs (MMP-2 or -9) either in the tumor matrix or surrounding cells in the microenvironment is likely to contribute to different invasive ability. This hypothesis is further bolstered by the notion that MMPs alone do not contribute much to invasion or migration of tumor cells (Figure S2D).

What is the mechanism by which MMPs secreted by astrocytes trigger invasion of tumor cells? MMP-2 and -9 secreted by leukemic cells have been shown to increase the permeability of BBB by disrupting tight junction proteins [38]. Similarly, in local ischemia in rat, synthetic MMP inhibitors reverse the MMP-mediated disruption of tight junction proteins in cerebral vessels [39]. These studies imply that secreted MMPs are capable of disrupting BBB thus facilitating invasion into the brain microenvironment. In our study, astrocyte-conditioned tumor cells were injected into the heart. From this site, the tumor cell enters circulation and travels via organs (ex: lung) en route to brain or directly accesses the brain microenvironment. Either way, the astrocyte-conditioned tumor cells hone to the brain earlier than non-conditioned tumor cells or cell previously conditioned for brain homing. Because astrocyte-conditioned tumor cells show invasive phenotype, it suggests morphological changes in tumor cells (LW, RR unpublished data) that influence its ability to invade and metastasize. Alternatively, latent MMP substrates on tumor cells or cells of the tumor microenvironment may be activated upon cleavage by astrocyte secreted MMPs resulting in an invasive phenotype. Because breast cancer and lung cancer WT cells are already expressing MMP-2 and MMP-9 (Figure S6, See File S1), and assuming RNA levels translates into active protein levels, these cells should be primed for invasion but they are not. Therefore, we favor the morphological alteration model that is dictated in part by genomic changes in the astrocyte-conditioned tumor cells, which is currently under investigation in our laboratory.

**Figure 7:**
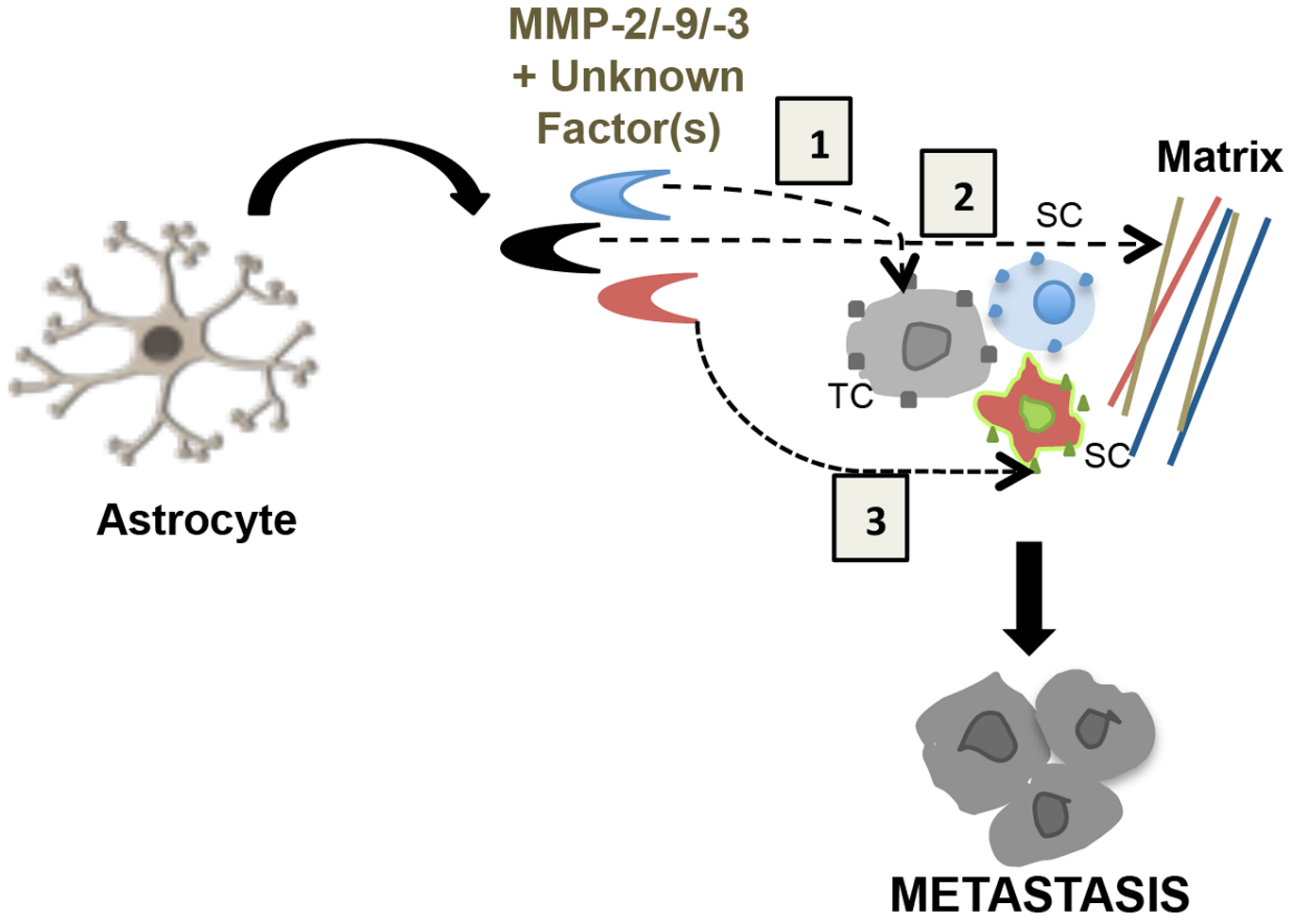
Working model for the effects of astrocyte secreted MMP-2/-9/-3 on tumor metastasis. In this model, astrocyte-secreted MMPs and unknown factor(s) induce cleavage of substrates on tumor cell or surrounding cells in the microenvironment that leads to tumor invasion and metastasis. TC: tumor cell; SC: surrounding cell.

The outcome of the metastatic process depends on productive interactions of tumor cells with host homeostatic mechanisms [40], [41]. In the brain, such productive interactions for tumor cells are likely to occur with astrocytes. Although reactive astrocytes surrounding brain metastases has been observed [4], [8] our data suggests a novel mechanism by which tumor cells when conditioned with astrocyte secretome (MMP-2 and -9) becomes highly migratory and invasive leading to tumor cells metastasis to the brain and other sites (Figure 7). We provide evidence for a direct role astrocytes play in altering the immediate tumor microenvironment to facilitate tumor cell invasion leading to increased metastasis to the brain. This study collectively suggests a direct link between astrocytes and tumor progression, and that astrocytes directly influence tumor growth and metastasis.

## Supporting Information

Figure S1 **Determine astrocyte-secreted components that mediate tumor cell migration and invasion.** (A) Effect of EET-EZ on S180/H2030 cell migration. Boyden chamber assay was performed for 5 h with S180 cells and H2030 cells in astrocyte CM (Ast^CM^), or astrocyte CM pre-treated with 10 µM of EETs antagonist EET-EZ (Ast^CM-EZ^). *p<0.05 compared with DMEM; NS: no significance. (B) & (C) The effects of heparin binding proteins on S180 cell migration and invasion. S180 cell migration and invasion were examined using the wound healing assay (B, 7 h) and invasion assay (C, 8 h) with astrocyte CM (Ast^CM^) or astrocyte CM pre-incubated with heparin agarose beads (Ast^CM-Hep^) for 3 h. *p<0.05 compared with DMEM. NS: no significance; (D) S180 cell invasion in response to astrocyte ultracentrifuge fractionation elutes. Astrocyte CMs from ultrafiltration cut-off (Ast^CM-F^) were used for S180 cell invasion assay (8 h). Upper panel display invaded cells on the lower surface of the filter; Lower panel is the quantity of the CMs-induced tumor cell invasion. *p<0.01 compared with DMEM. For A, C and D, values are mean ± SD, n≥3.
(PDF)

Figure S2 **Effects of MMP-2, MMP-9, and MMP-3 protein modulations and activity inhibition on astrocyte secretome-induced cancer cell migration and invasion.** (A) Effects of MMP inhibitors on astrocyte CM-induced S180 cell migration. Wound healing assay (7 h) was performed in S180 with astrocyte CM (Ast^CM^) pre-treated with MMP inhibitors ONO-4817, Batimastat and Marimastat; (B) ONO-4817 inhibits S180 cell invasion in a dose-dependent pattern. Astrocyte CM pre-treated with different dosage of ONO-4817 was used for S180 invasion assay (8 h). *p<0.05 and **p<0.01 compared with only astrocyte CM (Ast^CM^). Values are mean ± SD, n≥3. (C) MMP-3 is involved in astrocyte CM-induced MDA-MB-231 cell invasion. Astrocyte CM pre-pulled down with anti-MMP-2, anti-MMP-3 and anti-MMP-9, respectively. The resulted medium was submitted to invasion assay (14 h). *p<0.05 and **p<0.01 compared with only astrocyte CM (Ast^CM^). Values are mean ± SD, n≥3. (D) MMPs do not possess chemoattractive properties. Purified human MMP-2, MMP-3 and MMP-9 proteins were added to the lower chamber for MDA-MB-231 cell invasion assay (14 h).
(PDF)

Figure S3 **Astrocyte CM-induced breast cancer metastasis formation.** Images show all mice in the three groups under study. Group A: WT MDA-MB-231P5D-Luc cells, n = 7; group B: astrocyte CM-induced breast cancer MDA-MB-231P5A-Luc cells, n = 8; group C: Brain homing MDA-MB-231Br cells, n = 6.
(PDF)

Figure S4 **MMP-2 and MMP-9 are involved in astrocyte CM-induce breast cancer brain metastasis formation.** (A) Images show all mice in the three groups under study. Group A: MDA-MB-231-P5D-Luc, n = 8; group B: MDA-MB-231P5A-Luc, n = 10; and group C: MDA-MB-231P5A/ONO4817-Luc, n = 10; (B) The normalized photon flux.
(PDF)

Figure S5 **Effects of astrocyte CM on non-brain metastatic tumor cell invasion.** Matrigel invasion assays were performed with colon HCT116 and breast MCF-7 cells, respectively, for 14 h. MDA-MB-231 cells were used as control. *p<0.01, values are mean ± SD, n≥3.
(PDF)

Figure S6 **Tumor cells secrete MMP-2 and MMP-9.** (A) qPCR of *MMP9* transcript in MDA-MB-231 human breast cancer cells untreated (WT) or exposed to astrocyte-conditioned media (Ast^CM^) for a duration of 5 passages. *p<0.01. Values are mean ± SD, n = 3; (B) H2030 cells and MDA-MB-231 cells were cultured in basal medium without FBS. The resulted medium was applied to western blotting to analyze tumor cell-secreted MMP-2 and MMP-9 proteins.
(PDF)

Table S1 A list of astrocyte-secreted proteins from the published literature is provided as a reference.
(PDF)

Table S2 Astrocyte secretome-induced tumor metastasis.
(PDF)

Table S3 Effects of MMP-2/-9 on astrocyte secretome-induced tumor metastasis.
(PDF)

File S1 Supporting materials and methods information has been provided in File S1.
(DOCX)
