# Correction: Astrocytes Directly Influence Tumor Cell Invasion and Metastasis *In Vivo*

**DOI:** 10.1371/journal.pone.0137369

**Published:** 2015-08-31

**Authors:** Ling Wang, Stephanie M. Cossette, Kevin R. Rarick, Jill Gershan, Michael B. Dwinell, David R. Harder, Ramani Ramchandran

The following information is missing from the Funding section: David Harder was supported by a Clement J. Zablocki VA Medical Center Career Research Scientist Award.
