# Supplementary figures and images for: Astrocytes Directly Influence Tumor Cell Invasion and Metastasis In Vivo
Source: PLoS One. 2013 Dec 4;8(12):e80933. doi: 10.1371/journal.pone.0080933 (PMC3851470; doi:10.1371/journal.pone.0080933)

# Figure S1

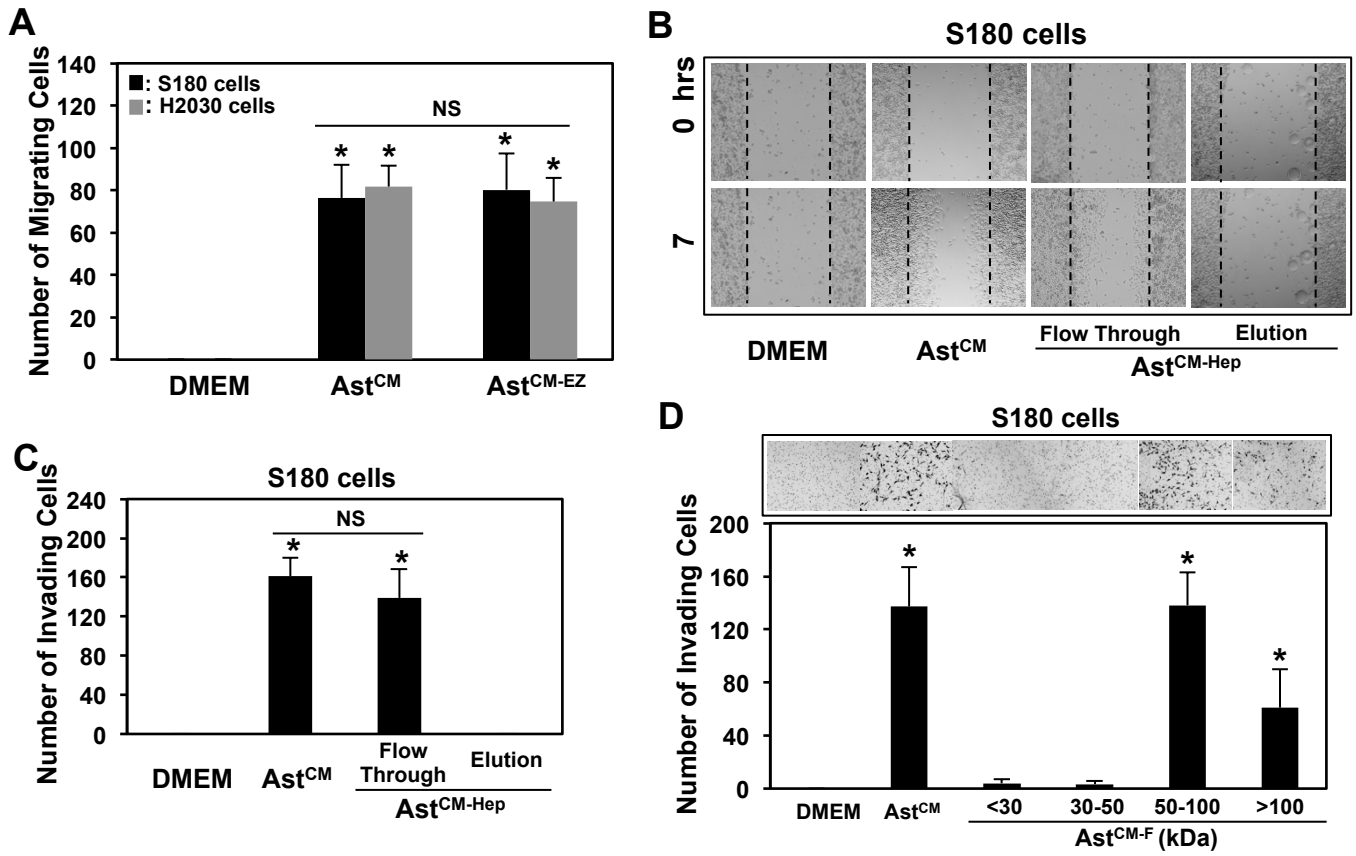

Supplement: Figure S1 — Determine astrocyte-secreted components that mediate tumor cell migration and invasion. (A) Effect of EET-EZ on S180/H2030 cell migration. Boyden chamber assay was performed for 5 h with S180 cells and H2030 cells in astrocyte CM (AstCM), or astrocyte CM pre-treated with 10 µM of EETs antagonist EET-EZ (AstCM-EZ). *p<0.05 compared with DMEM; NS: no significance. (B) & (C) The effects of heparin binding proteins on S180 cell migration and invasion. S180 cell migration and invasion were examined using the wound healing assay (B, 7 h) and invasion assay (C, 8 h) with astrocyte CM (AstCM) or astrocyte CM pre-incubated with heparin agarose beads (AstCM-Hep) for 3 h. *p<0.05 compared with DMEM. NS: no significance; (D) S180 cell invasion in response to astrocyte ultracentrifuge fractionation elutes. Astrocyte CMs from ultrafiltration cut-off (AstCM-F) were used for S180 cell invasion assay (8 h). Upper panel display invaded cells on the lower surface of the filter; Lower panel is the quantity of the CMs-induced tumor cell invasion. *p<0.01 compared with DMEM. For A, C and D, values are mean ± SD, n≥3. (PDF) [file pone.0080933.s001.pdf]

# Figure S2

**A**

S180 cells/Ast<sup>CM</sup>

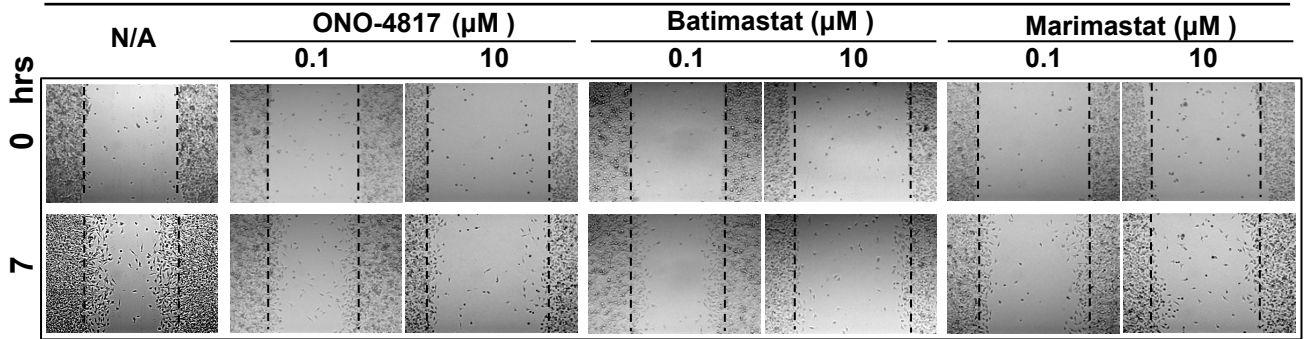

**B**

S180 cells

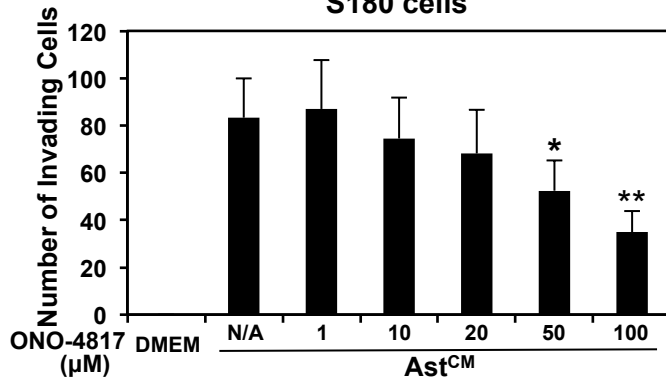

**C**

MDA-MB-231 cells

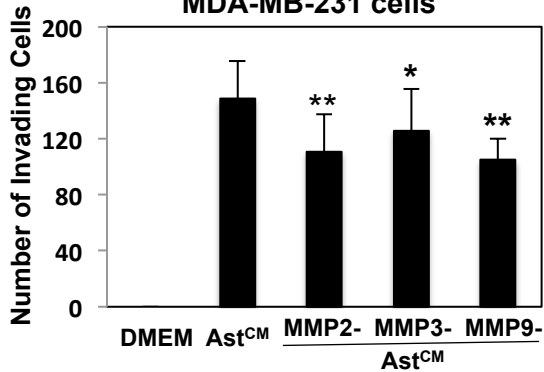

**D**

MDA-MB-231 cells

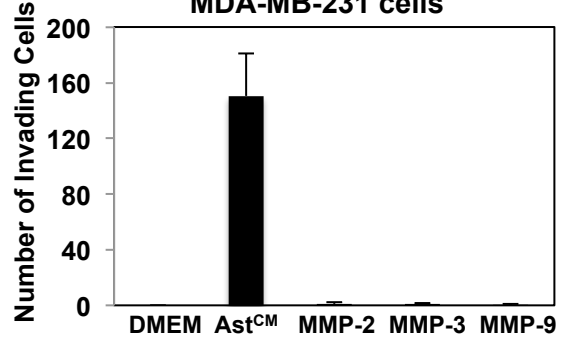

Supplement: Figure S2 — Effects of MMP-2, MMP-9, and MMP-3 protein modulations and activity inhibition on astrocyte secretome-induced cancer cell migration and invasion. (A) Effects of MMP inhibitors on astrocyte CM-induced S180 cell migration. Wound healing assay (7 h) was performed in S180 with astrocyte CM (AstCM) pre-treated with MMP inhibitors ONO-4817, Batimastat and Marimastat; (B) ONO-4817 inhibits S180 cell invasion in a dose-dependent pattern. Astrocyte CM pre-treated with different dosage of ONO-4817 was used for S180 invasion assay (8 h). *p<0.05 and **p<0.01 compared with only astrocyte CM (AstCM). Values are mean ± SD, n≥3. (C) MMP-3 is involved in astrocyte CM-induced MDA-MB-231 cell invasion. Astrocyte CM pre-pulled down with anti-MMP-2, anti-MMP-3 and anti-MMP-9, respectively. The resulted medium was submitted to invasion assay (14 h). *p<0.05 and **p<0.01 compared with only astrocyte CM (AstCM). Values are mean ± SD, n≥3. (D) MMPs do not possess chemoattractive properties. Purified human MMP-2, MMP-3 and MMP-9 proteins were added to the lower chamber for MDA-MB-231 cell invasion assay (14 h). (PDF) [file pone.0080933.s002.pdf]

**Figure S3**

**A**

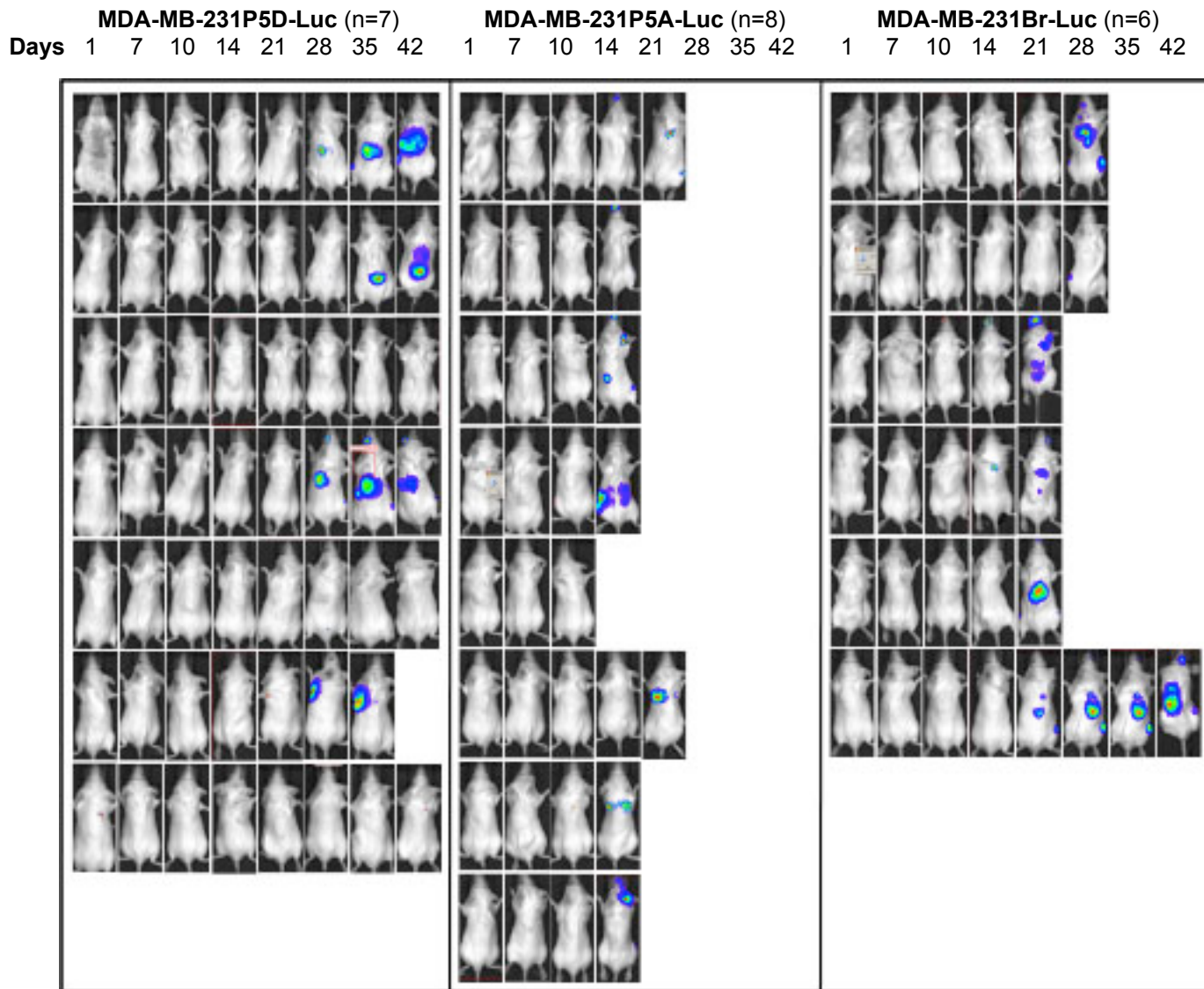

Supplement: Figure S3 — Astrocyte CM-induced breast cancer metastasis formation. Images show all mice in the three groups under study. Group A: WT MDA-MB-231P5D-Luc cells, n = 7; group B: astrocyte CM-induced breast cancer MDA-MB-231P5A-Luc cells, n = 8; group C: Brain homing MDA-MB-231Br cells, n = 6. (PDF) [file pone.0080933.s003.pdf]

# Figure S4

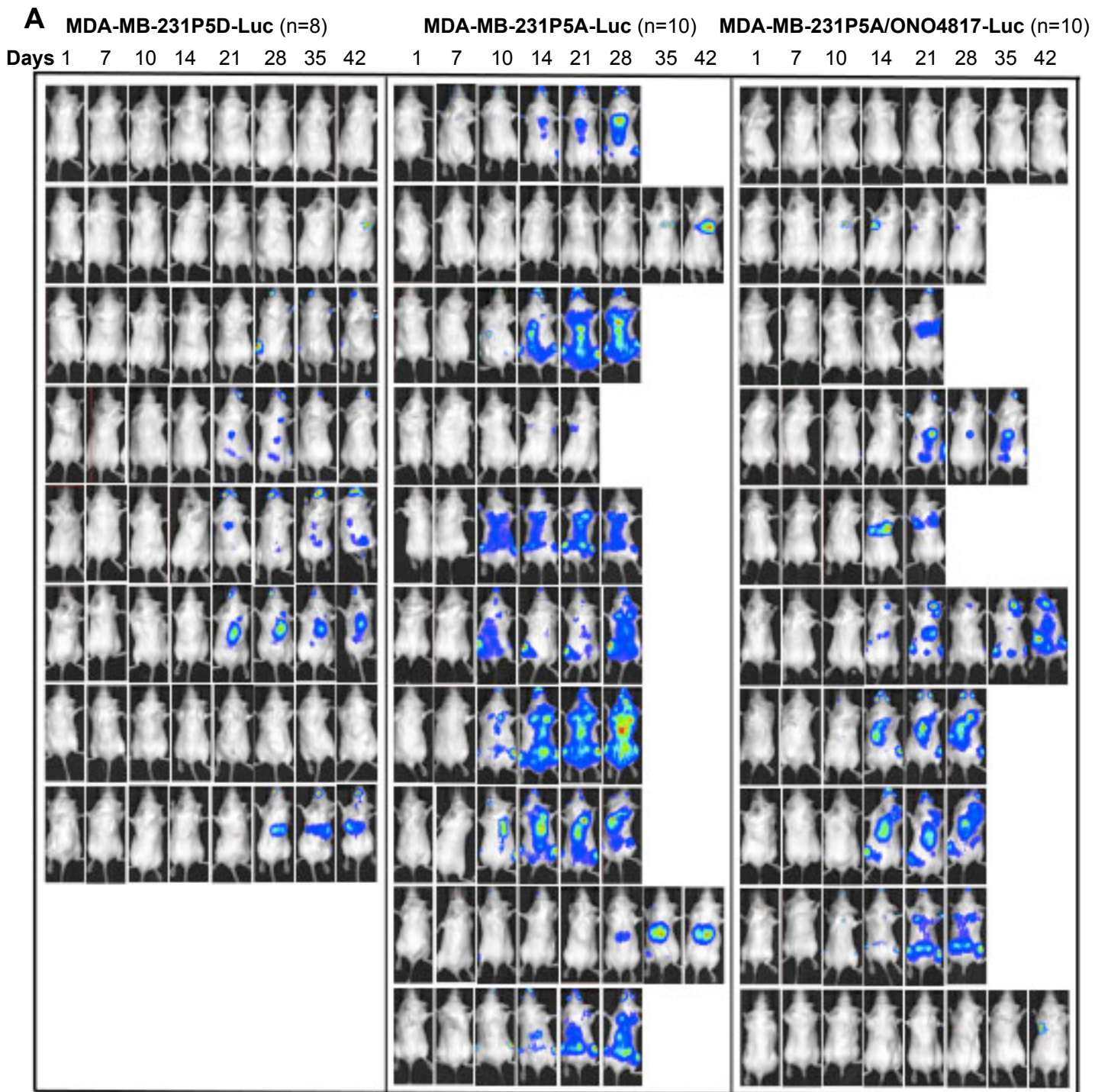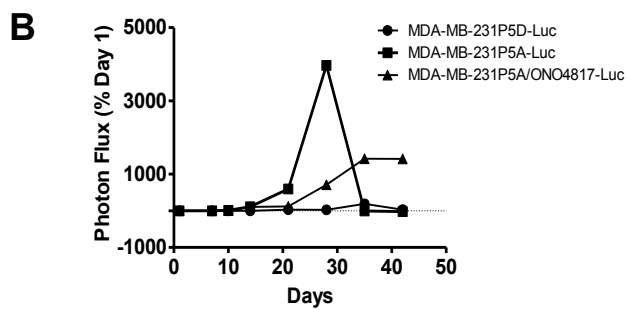

Supplement: Figure S4 — MMP-2 and MMP-9 are involved in astrocyte CM-induce breast cancer brain metastasis formation. (A) Images show all mice in the three groups under study. Group A: MDA-MB-231-P5D-Luc, n = 8; group B: MDA-MB-231P5A-Luc, n = 10; and group C: MDA-MB-231P5A/ONO4817-Luc, n = 10; (B) The normalized photon flux. (PDF) [file pone.0080933.s004.pdf]

**Figure S5**

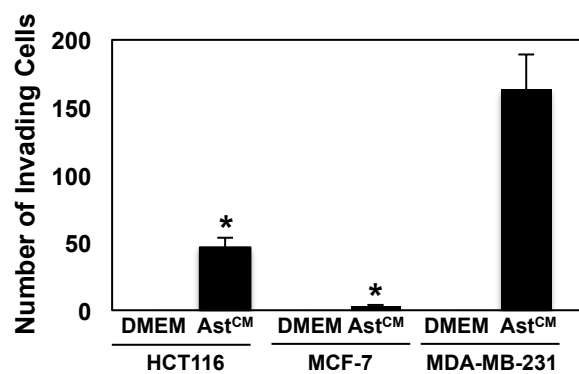

Supplement: Figure S5 — Effects of astrocyte CM on non-brain metastatic tumor cell invasion. Matrigel invasion assays were performed with colon HCT116 and breast MCF-7 cells, respectively, for 14 h. MDA-MB-231 cells were used as control. *p<0.01, values are mean ± SD, n≥3. (PDF) [file pone.0080933.s005.pdf]

# Figure S6

**A**

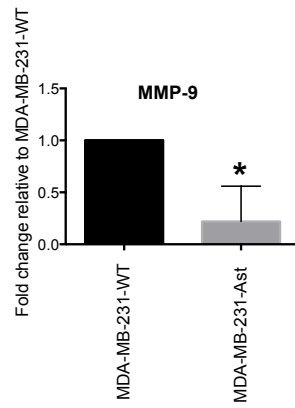

**B**

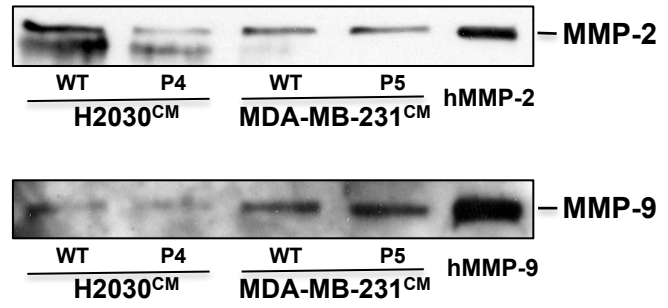

Supplement: Figure S6 — Tumor cells secrete MMP-2 and MMP-9. (A) qPCR of MMP9 transcript in MDA-MB-231 human breast cancer cells untreated (WT) or exposed to astrocyte-conditioned media (AstCM) for a duration of 5 passages. *p<0.01. Values are mean ± SD, n = 3; (B) H2030 cells and MDA-MB-231 cells were cultured in basal medium without FBS. The resulted medium was applied to western blotting to analyze tumor cell-secreted MMP-2 and MMP-9 proteins. (PDF) [file pone.0080933.s006.pdf]
