# Supplementary material for: Astrocytes Directly Influence Tumor Cell Invasion and Metastasis In Vivo
Source: PLoS One. 2013 Dec 4;8(12):e80933. doi: 10.1371/journal.pone.0080933 (PMC3851470; doi:10.1371/journal.pone.0080933)
Supplement: Table S1 — A list of astrocyte-secreted proteins from the published literature is provided as a reference. (PDF) [file pone.0080933.s007.pdf]

Table S1

Table S1. Molecular Weight of identified astrocyte-secreted chemokines

Molecular Weight (kDa)	< 50	50-100	> 100
Chemokines	IL-1β, IL-6, IL-8, IL-10 IL-17, IL-27, TNF-α, TGF-α, TGF-β, BMP2. BMP4, Noggin, BMPRIA, BMPRIB, BMPRII, IFN-γ17, IFN-β, CCL2 , CCL23, CCL5, CXCL10, CXCL12, Lipocalin-2, VEGF Fibronectin. 6RANTES, BDNF, GFAP, IGF.	MMP9, MMP3, MMP2.	Nestin.
