# Supplementary material for: Astrocytes Directly Influence Tumor Cell Invasion and Metastasis In Vivo
Source: PLoS One. 2013 Dec 4;8(12):e80933. doi: 10.1371/journal.pone.0080933 (PMC3851470; doi:10.1371/journal.pone.0080933)
Supplement: Table S2 — Astrocyte secretome-induced tumor metastasis. (PDF) [file pone.0080933.s008.pdf]

Table S2. Astrocyte secretome-induced tumor metastasize

Group	Mouse	Tumor	Brain Metastasis	Survival
MDA-MB-231P5D-Luc	7	4	1	6
MDA-MB-231P5A-Luc	8	8	6	0
MDA-MB-231Br-Luc	6	6	4	5
