# Supplementary material for: Astrocytes Directly Influence Tumor Cell Invasion and Metastasis In Vivo
Source: PLoS One. 2013 Dec 4;8(12):e80933. doi: 10.1371/journal.pone.0080933 (PMC3851470; doi:10.1371/journal.pone.0080933)
Supplement: Table S3 — Effects of MMP-2/-9 on astrocyte secretome-induced tumor metastasis. (PDF) [file pone.0080933.s009.pdf]

Table S3. Effects of MMP-2/-9 on astrocyte secretome-induced tumor metastasis

Group	Mouse	Tumor	Brain Metastasis	Survival
MDA-MB-231P5D-Luc	8	6	5	10
MDA-MB-231P5A-Luc	10	10	8	2
MDA-MB-231P5A/ ONO4817-Luc	10	9	6	3
