# Supplementary material for: Astrocytes Directly Influence Tumor Cell Invasion and Metastasis In Vivo
Source: PLoS One. 2013 Dec 4;8(12):e80933. doi: 10.1371/journal.pone.0080933 (PMC3851470; doi:10.1371/journal.pone.0080933)
Supplement: File S1 — Supporting materials and methods information has been provided in File S1. (DOCX) [file pone.0080933.s010.docx]

**Supporting Materials and Methods**

*Bromodeoxyuridine (BrdU) assay*

Cell proliferation was measured using an ELISA BrdU assay kit (Roche Molecular Biochemicals, Mannheim, Germany) was in accordance with the manufacturer’s protocol. Briefly, cells were cultured in 96-well plates at 5,000 cells per well and allowed to grow in astrocyte CM or DMEM for 24 h. Cells were then incubated with BrdU labeling reagent for 2 h. Then the cells were fixed and BrdU incorporation into newly synthesized DNA was assessed by incubation with an anti-BrdU peroxidase-conjugated antibody for 90 min, followed by addition of substrate solution and colorimetric detection at 405 nm with a reference wavelength at approximately 690 nm.

*Gelatin zymography*

Briefly, astrocyte CM was denatured in the absence of a reducing agent and electrophoresed in 10% SDS-PAGE containing 0.1% (w/v) gelatin. Gels were incubated in the presence of 2.5% Triton X-100 at room temperature for 2 h and subsequently at 37°C overnight in a buffer containing 10 mM CaCl_2_, 0.15 M NaCl, and 50 mM Tris (pH 7.5), and gels were stained with 0.25% Coomassie blue. Gelatinase activity was visualized as clear bands against the blue-stained gelatin background and analyzed in a computer system.

*qPCR*

Tumor cells were suspended in Trizol® and immediately frozen at -80°C. RNA was processed according to the Trizol® reagent manufacturer’s protocol (Invitrogen). The RNA optical density and ratio of absorbance at 260 and 280 nm (A_260/280_) was determined using a Nanodrop-1000 spectrophotometer (Thermo Scientific, Wilmington, DE)**.** cDNA was synthesized from 1-2 μg of RNA using QuantiTect reverse transcription cDNA kit (Qiagen, Valencia, CA) per manufacturer’s protocol. cDNA (1 μL at a 1:10 dilution) was amplified in a 20 μL reaction volume in the CFX C1000 Real-time Thermal Cycler (Bio-Rad) with the primer sets listed in the 5’ to 3’: Human MMP-9 forward CTGAGAACCAATCTCACCGACAGG and reverse CGGGCAGGGACAGTTGCTTCTG; and human MMP-2 forward CCCAAAACGGACAAAGAGTTGG and reverse TGTGTCCTTCAGCACAAACAG. Primer R^2^ values and efficiencies (E) were as follows: Human MMP-9: R^2^=0.975, E=109.4% and human MMP-2: R^2^=0.972, E=115.8%. Reagents included the SsoFast™ EvaGreen® Supermix (Bio-Rad) using the following amplification protocols: 95°C for 30 seconds followed by 39 cycles of 95°C for 3 seconds and 60°C for 3 seconds. Samples were then heated to 95°C for 10 seconds followed by a melt curve from 65°C to 95°C with 0.5°C increments for 5 seconds. The 18s reference gene was amplified using QuantiTect 18s primers (Qiagen). All reactions were done in triplicate wells with duplicate well no template controls (NTC). cDNA from WT and astrocyte CM exposed cells was analyzed from 3 separate experiments. For each experiment, the fold change in gene expression was determined by calculating the average ΔΔCq values from triplicate wells and these values were combined from at least 3 separate qPCR amplifications.
